# Bortezomib sensitises TRAIL-resistant HPV-positive head and neck cancer cells to TRAIL through a caspase-dependent, E6-independent mechanism

**DOI:** 10.1038/cddis.2014.455

**Published:** 2014-10-23

**Authors:** J Bullenkamp, N Raulf, B Ayaz, H Walczak, D Kulms, E Odell, S Thavaraj, M Tavassoli

**Affiliations:** 1 Department of Molecular Oncology, King's College London, Guy's Campus, Hodgkin Building, London SE1 1UL, UK; 2 Department of Oral Pathology, King's College London, Guy's Campus, Dental Institute, London SE1 9RT, UK; 3 Centre for Cell Death, Cancer and Inflammation (CCCI), UCL Cancer Institute, 72 Huntley Street, London WC1E 6BT, UK; 4 Experimental Dermatology, Department of Dermatology, TU Dresden, Dresden 01307, Germany

## Abstract

Human papillomavirus (HPV) is causative for a new and increasing form of head and neck squamous cell carcinomas (HNSCCs). Although localised HPV-positive cancers have a favourable response to radio-chemotherapy (RT/CT), the impact of HPV in advanced or metastatic HNSCC remains to be defined and targeted therapeutics need to be tested for cancers resistant to RT/CT. To this end, we investigated the sensitivity of HPV-positive and -negative HNSCC cell lines to TRAIL (tumour necrosis factor-related apoptosis-inducing ligand), which induces tumour cell-specific apoptosis in various cancer types. A clear correlation was observed between HPV positivity and resistance to TRAIL compared with HPV-negative head and neck cancer cell lines. All TRAIL-resistant HPV-positive cell lines tested were sensitised to TRAIL-induced cell death by treatment with bortezomib, a clinically approved proteasome inhibitor. Bortezomib-mediated sensitisation to TRAIL was associated with enhanced activation of caspase-8, -9 and -3, elevated membrane expression levels of TRAIL-R2, cytochrome *c* release and G2/M arrest. Knockdown of caspase-8 significantly blocked cell death induced by the combination therapy, whereas the BH3-only protein Bid was not required for induction of apoptosis. XIAP depletion increased the sensitivity of both HPV-positive and -negative cells to TRAIL alone or in combination with bortezomib. In contrast, restoration of p53 following E6 knockdown in HPV-positive cells had no effect on their sensitivity to either single or combination therapy, suggesting a p53-independent pathway for the observed response. In summary, bortezomib-mediated proteasome inhibition sensitises previously resistant HPV-positive HNSCC cells to TRAIL-induced cell death through a mechanism involving both the extrinsic and intrinsic pathways of apoptosis. The cooperative effect of these two targeted anticancer agents therefore represents a promising treatment strategy for RT/CT-resistant HPV-associated head and neck cancers.

Head and neck squamous cell carcinoma (HNSCC) represents the sixth most common cancer worldwide.^1^ While the overall incidence of HNSCC, traditionally associated with tobacco or alcohol consumption, is declining, a subset of oropharyngeal cancers caused by infection with high-risk types of human papillomavirus (HPV) has risen significantly.^2,3^ Transformation upon HPV infection occurs mainly because of inactivation of the p53 and retinoblastoma tumour suppressor proteins mediated by the viral oncoproteins E6 and E7, respectively.^4^

HPV-positive (HPV^+^) cancers represent a distinct subset of HNSCC in terms of biology and clinical behaviour. In general, they are characterised by better overall survival and an improved response to conventional radio-chemotherapy (RT/CT) compared with HPV-negative (HPV^−^) cancers.^5,6^ To further minimise treatment-related toxicity without compromising outcome, there have been suggestions of treatment de-escalation in conjunction with targeted therapies.^7^

The novel anticancer agent TRAIL (tumour necrosis factor-related apoptosis-inducing ligand) selectively kills several types of malignant cell lines with little effect on normal cells.^8^ Recombinant TRAIL or monoclonal antibodies targeting TRAIL receptors (TRAIL-Rs) are currently being tested in phase I/II clinical trials for patients with advanced tumours.^9,10^ TRAIL induces cell death by binding to TRAIL-R1 or TRAIL-R2, resulting in receptor oligomerisation and formation of the death-inducing signalling complex (DISC)^11^ and activation of initiator caspase-8.^12^ Caspase-8 directly activates effector caspase-3 to induce apoptosis through the type I pathway or cleaves the BH3-only protein Bid, generating tBid. This type II pathway involves an amplification loop through the intrinsic pathway of apoptosis characterised by cytochrome *c* release from the mitochondria, activation of initiator caspase-9 and ultimately caspase-3.^13^

Despite its tumour-selective activity, various cancer cell lines remain resistant to TRAIL, limiting the clinical potential of TRAIL-based monotherapies. Many recent studies focus on combination strategies with other agents to sensitise resistant cells to TRAIL.^14^ The proteasome inhibitor bortezomib is an FDA-approved drug for the treatment of multiple myeloma, but has shown only little single-agent activity in solid malignancies such as HNSCC while being effective in combination with other treatment options.^15, 16, 17^ Combining bortezomib with TRAIL-R agonists produced a synergistic cytotoxic effect in various types of cancers. Potential mechanisms underlying sensitisation to TRAIL-induced apoptosis include inhibition of NF-*κ*B signalling, stabilisation of BH3-only proteins, p53 or p21, upregulation of TRAIL-Rs and enhanced stability of caspase-8.^18, 19, 20, 21, 22, 23, 24, 25, 26^

So far, little data is available on the therapeutic potential of TRAIL alone or in combination with bortezomib in HNSCC or other HPV^+^ related cancers. Treatment with the proteasome inhibitor MG132 sensitised TRAIL-resistant HPV^+^ cervical cancer cells to TRAIL through p53-dependent upregulation of TRAIL-Rs and inactivation of XIAP.^27^ Overexpression of E6 was shown to protect colon cancer cells from death receptor-induced apoptosis by affecting the stability of the DISC, indicating a functional link between the presence of E6 and TRAIL signalling.^28^

In this study, we tested the response of HPV^+^ and HPV^−^ HNSCC cells to treatment with TRAIL alone or combined with bortezomib, revealing a clear pattern of sensitivity to TRAIL depending on HPV status and a synergistic effect when combined with bortezomib. In addition, we identified some of the proteins and pathways involved in the response to TRAIL/bortezomib in HNSCCs.

## Results

### HPV-associated HNSCC cells are resistant to TRAIL

**Figure 1:**
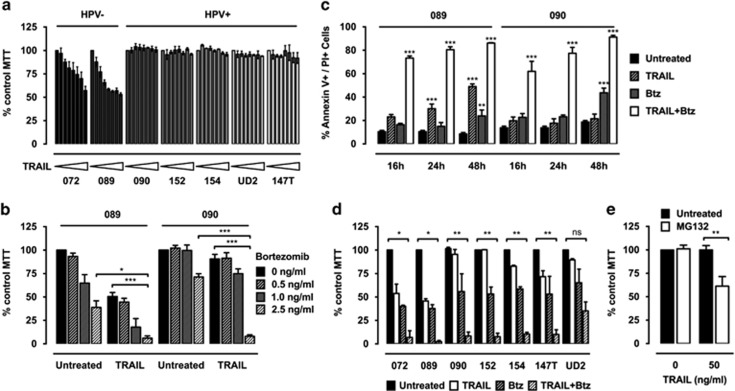
Sensitisation of HPV^+^ HNSCC cells to TRAIL by proteasome inhibition. (**a**) Seven HPV^−^ or HPV^+^ HNSCC cell lines were treated with increasing concentrations of TRAIL up to 100 ng/ml for 72 h. Cell viability after treatment was analysed by 3-(4,5-dimethythiazol-2-yl)-2,5-diphenyl tetrazolium bromide (MTT) assay of triplicate wells. Bars represent mean cell viability normalised to untreated control cells and error bars indicate S.E.M. of three independent experiments. (**b**) The 089 and 090 cells were treated with the indicated concentrations of TRAIL and/or bortezomib (Btz) for 48 h and analysed by MTT assay as before in four independent experiments. *P*-values were determined by two-way analysis of variance (ANOVA) (*P<0.05 and ***P<0.001). (**c**) The 089 and 090 cells were treated with 50 ng/ml TRAIL and 2.5 ng/ml Btz alone or in combination. Cells were collected at the indicated time points for Annexin V/PI staining and fluorescence-activated cell sorting (FACS) analysis. Bars indicate the mean percentage of dead cells defined as either Annexin V or PI positive and error bars indicate S.E.M. of three independent experiments. *P*-values were determined by two-way ANOVA (**P<0.01 and ***P<0.001). (**d**) Seven HNSCC cell lines were treated with single drugs or the combination of 50 ng/ml TRAIL and 2.5 ng/ml bortezomib for 48 h and analysed by MTT assay as before in three independent experiments. *P*-values were determined by two-way ANOVA (*P<0.05, **P<0.01, ^NS^*P*>0.05). (**e**) The 090 cells were treated with 50 ng/ml TRAIL and 0.1 *μ*M MG132 for 48 h until MTT analysis as before in three independent experiments. *P*-values were determined by two-way ANOVA (**P<0.01)

HPV^+^ HNSCCs generally display higher sensitivity to RT/CT than HPV^−^ cancers,^6^ but their response to targeted therapeutic agents remains largely unknown. We therefore investigated the cytotoxic effect of the tumour-selective apoptosis-inducing agent TRAIL in a panel of HPV^+^ and HPV^−^ HNSCC cell lines, demonstrating a link between HPV status and TRAIL sensitivity. Whereas treatment with increasing concentrations of recombinant TRAIL reduced the viability of HPV^−^ cells in a dose-dependent manner, all HPV^+^ cell lines tested remained resistant to TRAIL even at high concentrations (Figure 1a).

### Bortezomib sensitises HPV^+^ HNSCC cells to TRAIL-induced cell death

The proteasome inhibitor bortezomib has been shown to sensitise cells to TRAIL in other types of cancer.^29^ We therefore aimed to investigate whether concentrations of bortezomib with only low single-agent toxicity synergise with TRAIL in HPV^+^ HNSCC cells. The representative site-matched HNSCC cell lines 089 (HPV^−^) and 090 (HPV^+^), which demonstrated the highest difference in TRAIL sensitivity, were selected for further analysis. An initial dose–response assay revealed a similar response of 089 and 090 cells to treatment with bortezomib with an IC_50_ value of ~2.5 ng/ml (Supplementary Figure 1). The combined effect of both drugs was tested using 50 ng/ml TRAIL in combination with bortezomib at several concentrations below the IC_50_ value. Cotreatment with bortezomib sensitised HPV^+^ 090 cells to TRAIL-induced cell death, whereas bortezomib alone did not markedly impair cell viability (Figure 1b). This effect was highly synergistic with a combination index below 0.5 (data not shown). In addition, TRAIL-induced cell death in HPV^−^ 089 cells was further enhanced by bortezomib (Figure 1b).

The decrease in cell viability measured by MTT assays might also be because of bortezomib-induced cell cycle arrest reducing the number of viable cells. To determine whether combination treatment with TRAIL and bortezomib (TRAIL/bortezomib) triggers apoptosis in HNSCC cell lines, 089 and 090 cells were analysed by flow cytometry at the indicated time points (Figure 1c). Cell death, defined as positive staining for Annexin V or PI, was detected in both cell lines treated with TRAIL and bortezomib in combination. In contrast, TRAIL alone did not affect the viability of 090 cells but induced cell death in 089 cells, whereas bortezomib as a single drug displayed only slight toxicity in both cell lines.

A similar effect of bortezomib on TRAIL-induced cell death was also observed in a larger panel of HPV^+^ and HPV^−^ HNSCC cell lines. Cotreatment with TRAIL and bortezomib significantly reduced cell viability in all HPV^+^ cell lines compared with single treatments (Figure 1d). Overall, these results suggest that TRAIL-resistant HPV^+^ HNSCC cells become highly sensitive to TRAIL to a similar level as HPV^−^ cells when additionally treated with bortezomib.

To examine whether the sensitising effect of bortezomib is due to proteasome inhibition, another proteasome inhibitor, MG132, was tested in combination with TRAIL. Similar to bortezomib, cotreatment with a subtoxic dose of MG132 sensitised HPV^+^ 090 cells to TRAIL-induced cell death (Figure 1e).

### TRAIL and bortezomib combination treatment induces caspase-dependent cell death

**Figure 2:**
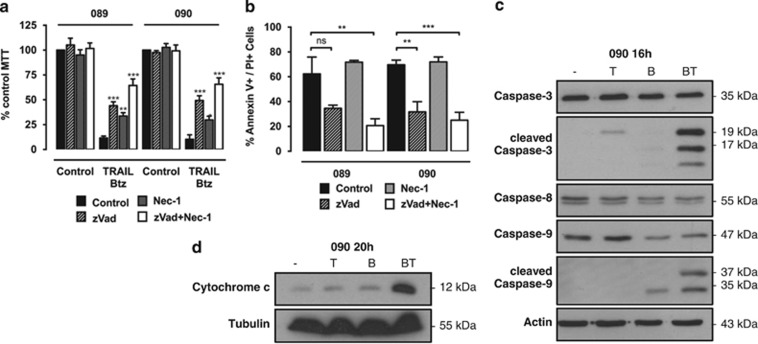
TRAIL and bortezomib cooperate to induce caspase-dependent apoptosis. (**a**) The 089 and 090 cells were treated with 50 ng/ml TRAIL and 2.5 ng/ml BTZ alone or in combination with the caspase inhibitor zVad (20 *μ*M) and/or the RIP1 kinase inhibitor nec-1 (50 *μ*M). Cell survival was measured 24 h later by 3-(4,5-dimethythiazol-2-yl)-2,5-diphenyl tetrazolium bromide (MTT) analysis. Bars represent mean cell viability normalised to untreated control cells and error bars indicate S.E.M. of four independent experiments. *P*-values were determined by two-way ANOVA (*P<0.05, **P<0.01 and ***P<0.001). (**b**) Quantification of Annexin V and PI single- or double-positive populations in 089 and 090 cells treated with 50 ng/ml TRAIL and 2.5 ng/ml bortezomib combined with zVad and/or nec-1 as before. Bars represent the percentage of dead cells that were stained positive for Annexin V, PI or both and error bars indicate S.E.M. of three independent experiments. *P*-values were determined by one-way ANOVA (**P<0.01 and ***P<0.001). (**c**) Western blot analysis of caspase-3, -8 and -9 processing in 090 cells treated with TRAIL (T, 50 ng/ml) and bortezomib (B, 2.5 ng/ml) alone or in combination (BT) for 16 h. (**d**) Cytochrome *c* release was analysed by western blot analysis of cytosolic fractions from 090 cells treated with TRAIL (T, 50 ng/ml) and bortezomib (B, 2.5 ng/ml) alone or in combination (BT) for 20 h

In addition to apoptosis TRAIL has also been implicated in necroptotic cell death.^30^ To analyse the type of cell death, 089 and 090 cells were pre-treated with the pancaspase inhibitor z-Vad-fmk (zVad) and/or the RIP1 kinase inhibitor necrostatin-1 (nec-1) followed by treatment with TRAIL/bortezomib. Pre-treatment with zVad significantly rescued both cell lines from TRAIL/bortezomib-induced cell death, increasing cell viability by ~30%. Further combination treatment with nec-1 provided significant additional protection by about 20%, which might indicate a necroptotic cell death induced by TRAIL/bortezomib in HNSCC cells (Figure 2a). These results were confirmed by Annexin V/PI staining after treatment of 089 and 090 cells with TRAIL/bortezomib plus zVad and/or nec-1. Addition of zVad alone or combined with nec-1 had a protective effect in both cell lines, reducing cell death by up to 40% (Figure 2b), confirming that TRAIL/bortezomib induce caspase-dependent cell death.

The activation of specific caspases in HPV^+^ 090 cells in response to the combination treatment was further analysed. Marked processing of caspase-3, generating the active 17 kDa fragment, and a slight reduction of procaspase-8 levels were only detected following treatment with TRAIL/bortezomib but not TRAIL alone (Figure 2c). Combination treatment but not individual drugs induced activation of caspase-9 as shown by the reduction in full-length caspase-9 levels and generation of the active 37 kDa fragment. This suggests activation of the intrinsic pathway of apoptosis, which is characterised by the release of cytochrome *c* from the mitochondria into the cytosol.^31^ Cytochrome *c* was detected in cytosolic fractions of 090 cells following combination treatment with TRAIL and bortezomib, hinting towards an involvement of the intrinsic pathway (Figure 2d).

### Bortezomib-mediated sensitisation to TRAIL is associated with upregulation of TRAIL-R2 and requires caspase-8 but not Bid

**Figure 3:**
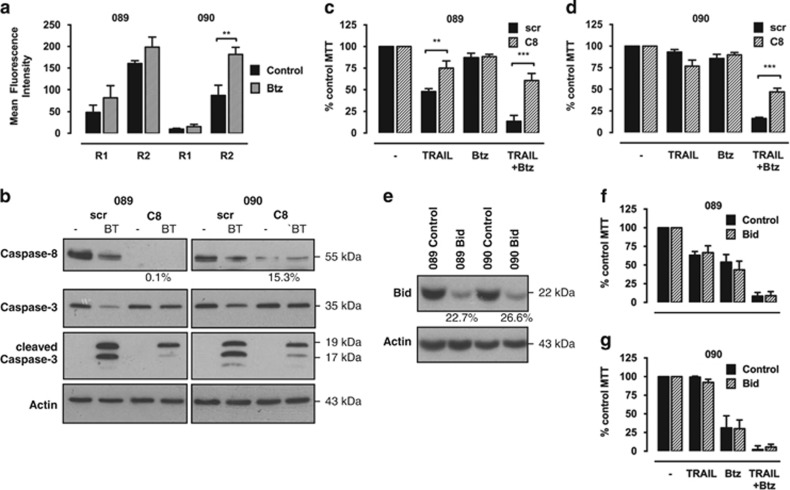
Bortezomib-induced sensitisation involves upregulation of DR5 and is mediated by caspase-8 but not Bid. (**a**) The 089 and 090 cells were treated with 2.5 ng/ml bortezomib (Btz) for 20 h. The surface expression of TRAIL-Rs was assessed by flow cytometry following staining with monoclonal antibodies for TRAIL-R1 (DR4) or TRAIL-R2 (DR5) and a secondary anti-mouse fluorescein isothiocyanate (FITC)-coupled antibody. Bars represent mean fluorescence intensity of FITC corrected for staining with a nonspecific isotype control and error bars indicate S.E.M. of three independent experiments. *P*-values were determined by one-way analysis of variance (ANOVA) (**P<0.01). (**b**) Stable inducible 089 and 090 caspase-8 (C8) and scrambled control (scr) short hairpin RNA (shRNA) cells were generated by lentiviral infection and shRNAs were induced with 1 *μ*g/ml doxycycline for 24 h. Caspase-3 and -8 levels following treatment with 50 ng/ml TRAIL and 2.5 ng/ml bortezomib (BT) for 16 h were assessed by western blot analysis compared with respective control cells. The efficiency of caspase-8 knockdown was calculated using ImageJ software normalised to *β*-actin. Blots were cut and combined at the indicated line. (**c** and **d**) Cell viability of 089 (**c**) and 090 (**d**) C8 and scr shRNA cells after induction with 1 *μ*g/ml doxycycline in the presence of 50 ng/ml TRAIL and/or 1 ng/ml Btz was analysed by 3-(4,5-dimethythiazol-2-yl)-2,5-diphenyl tetrazolium bromide (MTT) assay after 48 h of treatment. Bars represent mean cell viability normalised to untreated control cells and error bars indicate S.E.M. of three independent experiments. *P*-values were determined by two-way ANOVA comparing C8 shRNA with corresponding control cells (**P<0.01 and ***P<0.001). (**e**) Stable Bid and non-silencing control shRNA 089 and 090 cells were generated by lentiviral infection. The efficiency of Bid knockdown in both cell lines was assessed by western blot analysis compared with respective control cells and calculated using ImageJ software normalised to *β*-actin. (**f** and **g**) Cell viability of 089 (**f**) and 090 (**g**) Bid and control shRNA cells in the presence of 50 ng/ml TRAIL and/or 1 ng/ml Btz as indicated was analysed by MTT analysis after 48 h. Bars represent mean cell viability normalised to untreated control cells and error bars indicate S.E.M. of three independent experiments

Proteasome inhibition has previously been associated with increased transcription and membrane expression of TRAIL-R2.^18,32^ We therefore analysed the surface expression levels of TRAIL-Rs in 089 and 090 cells by flow cytometry. Measurement of basal receptor levels showed expression of TRAIL-R2 in both cell lines, whereas TRAIL-R1 was only detectable in 089 cells (Figure 3a and Supplementary Figure 2). Bortezomib treatment triggered a modest but significant increase in expression levels of TRAIL-R2 but not TRAIL-R1 in 090 cells. This might contribute to the increased TRAIL sensitivity of 090 cells; however, further studies will be required to determine whether TRAIL-R upregulation is necessary for TRAIL sensitisation by bortezomib.

The major initiator caspase recruited to the activated TRAIL-R complex is caspase-8. Knockdown of caspase-8 in 089 and 090 cells (knockdown efficiency 80–100%) markedly reduced processing of caspase-3 in response to the combination treatment (Figure 3b). Moreover, it significantly increased cell survival in both cell lines following treatment with TRAIL/bortezomib by ~40% compared with control cells (Figures 3c and d and Supplementary Figure 3). The remaining cytotoxic effect could be due to residual levels of caspase-8 or activation of other initiator caspases compensating for caspase-8 deficiency.

The truncated form of Bid, tBid, links death receptor activation to the intrinsic pathway and is stabilised by proteasome inhibition, representing a potential mechanism for sensitisation to TRAIL by bortezomib.^25^ The effect of Bid depletion on the sensitivity to TRAIL/bortezomib was studied using stable 089 and 090 Bid shRNA cells (knockdown efficiency 70% Figure 3e). No significant difference in the response to TRAIL and/or bortezomib was observed between Bid knockdown and control cells (Figures 3f and g), indicating that bortezomib-induced sensitisation to TRAIL occurs independently of Bid.

### XIAP depletion increases cell death in response to TRAIL alone and in combination with bortezomib

**Figure 4:**
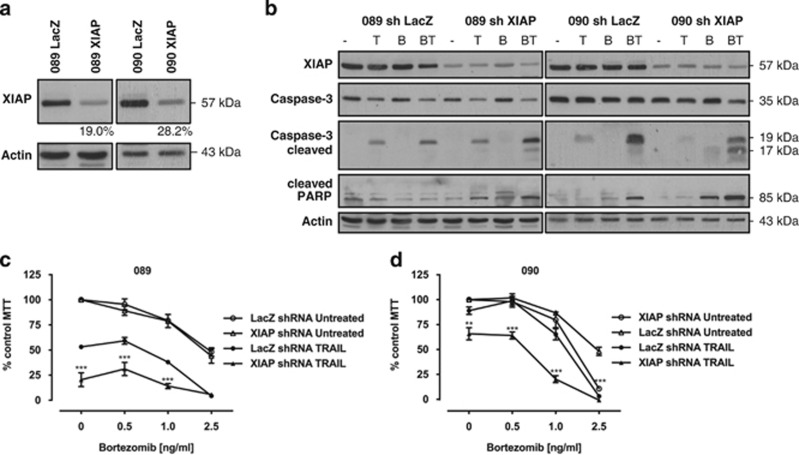
XIAP depletion enhances sensitivity of HPV^+^ cells to TRAIL alone or combined with bortezomib. (**a**) Stable XIAP and LacZ control shRNA 089 and 090 cells were generated by lentiviral infection. The efficiency of XIAP knockdown in both cell lines was assessed by western blot analysis compared with respective control cells and calculated using ImageJ software normalised to *β*-actin. Blots were cut and combined at the indicated line. (**b**) Stable 089 and 090 XIAP and LacZ short hairpin RNA (shRNA) cells were treated for 16 h with 50 ng/ml TRAIL alone (T), 2.5 ng/ml bortezomib (B) or both combined (BT). Protein levels of XIAP, caspase-3 and cleaved poly ADP ribose polymerase (PARP) were analysed by immunoblotting. (**c** and **d**) Cell viability of 089 (**c**) and 090 (**d**) XIAP and LacZ shRNA cells in the presence of 50 ng/ml TRAIL in combination with increasing bortezomib concentrations as indicated was analysed by 3-(4,5-dimethythiazol-2-yl)-2,5-diphenyl tetrazolium bromide (MTT) assay after 48 h. Bars represent mean cell viability normalised to untreated control cells and error bars indicate S.E.M. of four independent experiments. *P*-values were determined by two-way ANOVA comparing XIAP shRNA with corresponding LacZ control cells (**P<0.01 and *** *P*<0.001)

The antiapoptotic protein XIAP prevents caspase-3 activation and its inhibition has been linked to increased sensitivity to TRAIL.^33,34^ Stable knockdown of XIAP in 090 cells (knockdown efficiency 70%) resulted in enhanced caspase-3 activation in response to bortezomib alone or in combination with TRAIL, as evident by increased caspase-3 processing and cleavage of PARP, but did not markedly affect caspase-3 activation following treatment with TRAIL alone at this time point (Figure 4b). In contrast, stable 089 XIAP knockdown cells (knockdown efficiency 80%) showed increased processing and activity of caspase-3 in response to the combination treatment as well as TRAIL alone.

XIAP knockdown reduced the survival of 089 cells in response to TRAIL to a similar level as obtained in combination with bortezomib, but did not affect their sensitivity to bortezomib alone (Figure 4c). Importantly, TRAIL-resistant 090 cells were significantly sensitised to TRAIL following XIAP knockdown, which reduced cell viability in response to TRAIL alone by ~30% (Figure 4d). In both cell lines, depletion of XIAP significantly enhanced cell death after combination treatment with TRAIL and bortezomib at all concentrations tested.

### Bortezomib-induced p53 stabilisation in HPV^+^ cells is not the main mechanism for TRAIL sensitisation

**Figure 5:**
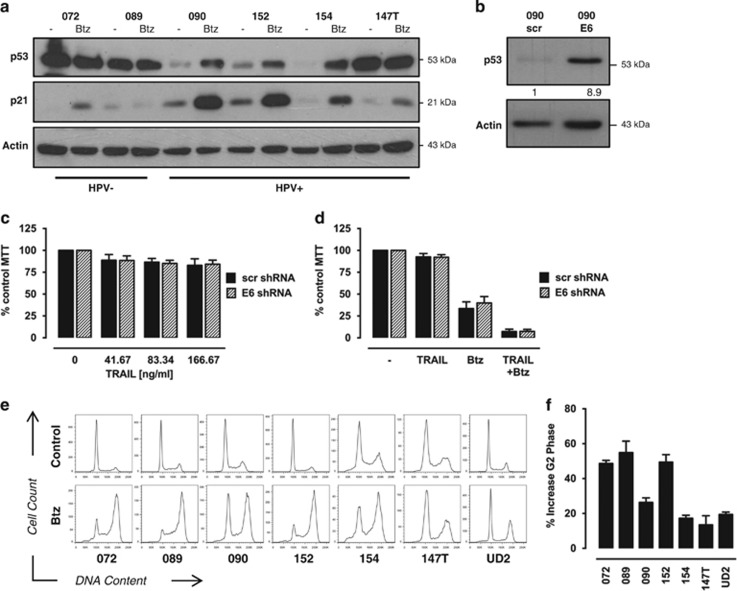
Role of p53 and E6 for bortezomib-mediated sensitisation to TRAIL. (**a**) The expression of p53 and p21 was detected by western blot analysis in six HNSCC cell lines after 16 h of treatment with 2.5 ng/ml bortezomib (Btz). (**b**) Stable E6 and scrambled control (scr) short hairpin RNA (shRNA) 090 cells were generated by lentiviral infection. E6 knockdown was confirmed by western blot analysis detecting p53 expression levels calculated using ImageJ software normalised to *β*-actin. (**c**) Cell viability of 090 E6 and scr shRNA cells treated with the indicated concentrations of TRAIL for 48 h was analysed by 3-(4,5-dimethythiazol-2-yl)-2,5-diphenyl tetrazolium bromide (MTT) assay of triplicate wells. Bars represent mean cell viability normalised to untreated control cells and error bars indicate S.E.M. of five independent experiments. (**d**) Cell viability of 090 E6 and scr shRNA cells in the presence of 50 ng/ml TRAIL alone or in combination with 2.5 ng/ml Btz was analysed by MTT assay after 48 h as before in five independent experiments. (**e**) Seven HNSCC cell lines were treated with 2.5 ng/ml Btz for 24 h before cell cycle analysis by flow cytometry. Representative histograms indicating cells in the respective cell cycle phases are shown. (**f**) Bars represent increase of the percentage of cells in the G2/M phase following treatment with 2.5 ng/ml bortezomib for 24 h compared with untreated control cells and error bars indicate S.E.M. of three independent experiments

In HPV^+^ tumours, p53 is kept inactive through E6-mediated proteasomal degradation,^35^ whereas HPV^−^ HNSCCs often contain inactivating p53 mutations, resulting in a stabilised but non-functional protein. Bortezomib treatment of HPV^+^ HNSCC cell lines has previously been associated with p53 stabilisation and induction of p53-dependent cell cycle arrest and apoptosis.^36^ Here we show that bortezomib induces restoration of p53 and induction of the p53 target protein p21 in all HPV^+^ cell lines tested with the exception of 147 T cells that already showed a marked basal p53 expression (Figure 5a). By contrast, HPV^−^ 089 and 072 cells, which contain mutated p53,^37^ showed no further increase in p53 protein levels and only a minimal induction of p21 in 072 cells.

To test the importance of p53 for the response of HPV^+^ cells to TRAIL/bortezomib, stable E6 shRNA 090 cells were generated. Western blot analysis showed almost ninefold increased levels of p53 in E6 knockdown cells compared with control cells, indicating reduced degradation of p53 after E6 depletion. E6 shRNA and control cells were subsequently treated with TRAIL alone or in combination with bortezomib for analysis of cell viability, but showed no significant difference in the response to either drug (Figures 5c and d). These results suggest that TRAIL resistance in 090 cells is not because of the presence and activity of E6 and that p53 restoration is insufficient to sensitise cells to TRAIL.

P21 represents a major mediator of p53-induced cell cycle arrest in the G0/G1 phase in response to DNA-damaging agents and other cellular stresses.^38^ Flow cytometry-based cell cycle analysis of bortezomib-treated HNSCC cells showed a strong G2/M arrest of up to 50% in all cell lines and a small increase in the sub-G1 population, indicating apoptotic cells (Figure 5e). The extent of G2/M arrest was more pronounced in HPV^−^ cells compared with HPV^+^ cells (Figure 5f), which might reflect their generally faster cellular proliferation rate (data not shown). However, the importance of this bortezomib-induced G2/M arrest for TRAIL-induced cell death in HNSCC cells requires further investigation.

## Discussion

HPV^+^ HNSCCs are characterised by improved overall and disease-specific survival compared with site- and age-matched controls.^5^ However, HPV status does not currently influence management of the disease and existing treatment modalities are associated with severe side effects. It is therefore necessary to investigate the impact of dose de-escalation and novel targeted therapeutic agents in this group of patients with the aim of maintaining efficacy while minimising toxicity. One such novel tumour-selective agent is TRAIL, and despite early promising result in other cancers,^8^ little is known about its effectiveness in HNSCC.

In contrast to the site-matched HPV^−^ cell lines tested here, HPV^+^ HNSCC cells were found to be highly resistant to TRAIL when used as a single agent even at high concentrations. There was no clear association between TRAIL resistance in HPV^+^ cells and endogenous levels of anti- or proapoptotic proteins such as TRAIL-Rs, caspase-3/-8 or XIAP (data not shown). However, expression profiling of a larger panel of regulatory proteins of apoptosis in a cohort of HPV^+^ and HPV^−^ HNSCC samples remains necessary to identify differentially expressed proteins that contribute to the distinct response pattern described here. From a clinical perspective, our data suggest that TRAIL is unlikely to be efficacious as a single modality treatment option for the management of HPV^+^ head and neck cancers.

The function of many proteins involved in death receptor signalling and apoptosis induction is regulated by ubiquitination and subsequent proteasomal degradation.^39^ In various other TRAIL-resistant cancer cells, including cell lines derived from squamous cell carcinomas, inhibition of proteasomal activity resulted in enhanced sensitivity to TRAIL-induced cell death.^22,23^ Here we show that combination treatment with recombinant TRAIL and low doses of the clinically approved proteasome inhibitor bortezomib displays synergistic cytotoxicity in all HPV^+^ HNSCC cell lines tested. While bortezomib treatment alone showed only a slight effect on the activation of apoptotic pathways, bortezomib markedly enhanced TRAIL-induced apoptosis in HNSCC cells, accompanied by increased processing of caspase-3, -8 and -9. A recent study demonstrated increased activity of recombinant TRAIL in patient-derived ovarian carcinoma cells when combined with bortezomib, while primary hepatocytes remained resistant, indicating low toxicity of the combination treatment in normal cells.^40^ The combined cytotoxic effect of bortezomib and TRAIL in both HPV^+^ and HPV^−^ cell lines suggests a promising novel therapeutic approach for the effective and selective killing of HNSCCs.

A number of preclinical studies in other types of cancer have identified mechanisms underlying the sensitisation of cancer cells to TRAIL by bortezomib. These include increased expression of TRAIL-Rs,^21,32^ enhanced activation of caspase-8,^26^ stabilisation of BH3-only proteins,^19,25^ inactivation of XIAP^27,34^ or induction of p53 or p21.^20,27^ Based on these studies, we aimed to investigate pathways that contributed to the increased sensitivity of HNSCC cells to TRAIL when combined with bortezomib.

Consistent with other studies, bortezomib treatment of HPV^+^ cells was associated with elevated membrane expression levels of TRAIL-R2.^18,32^ While this modest increase is unlikely to be the sole mechanism for the observed synergy, it may contribute to TRAIL sensitivity because of an increased number of receptors on the cell surface and therefore a higher degree of caspase-8 activation. Procaspase-8 levels were slightly reduced in HPV^+^ cells following combination treatment with TRAIL and bortezomib, suggesting enhanced activation of caspase-8 compared with single treatments. Moreover, bortezomib might also stabilise the active caspase-8 fragment to enhance its activity as suggested by recent reports that provided evidence for its regulation through ubiquitination and degradation.^41^ Depletion of caspase-8 resulted in markedly reduced processing of caspase-3 and provided significant protection against TRAIL/bortezomib in both HPV^+^ and HPV^−^ cells. Nevertheless, ~50% of cells remained sensitive to combination treatment with TRAIL/bortezomib in the absence of caspase-8, which might indicate a role for another initiator caspase such as caspase-10.

TRAIL and bortezomib in combination triggered cytochrome *c* release and activation of caspase-9, indicating induction of the mitochondrial pathway of apoptosis. The stability of the truncated form of the BH3-only protein Bid, which triggers the permeabilisation of the mitochondrial membrane after cleavage by active caspase-8, is regulated by proteasomal degradation.^42^ In glioblastoma cells, bortezomib-mediated stabilisation of tBid was shown to facilitate TRAIL-induced cell death by amplifying apoptosis through the mitochondrial pathway.^24,25^ In HNSCC cells, depletion of Bid had no effect on the cytotoxicity of TRAIL and bortezomib, suggesting that bortezomib-mediated sensitisation of HPV^+^ cells to TRAIL is independent of Bid and that TRAIL-induced apoptosis is mediated through the type I pathway. Activation of the intrinsic apoptotic pathway in response to the combination treatment, as evident by cytochrome *c* release and cleavage of caspase-9, potentially represents a parallel cellular process triggered by bortezomib but is unlikely to be the decisive mechanism of cell death.^18^

XIAP prevents the activation of caspase-3 and -9 at different levels, including inhibition of substrate binding and proteasomal degradation of processed caspase-3 forms.^43^ Downregulation or inhibition of XIAP has been proposed as a mechanism of bortezomib-induced sensitisation to TRAIL in different cancer models.^34,44,45^ In our system, depletion of XIAP partially sensitised HPV^+^ cells to TRAIL and significantly enhanced the synergistic effect of bortezomib. Similar data were obtained in HPV^+^ cervical cancer cells in which XIAP downregulation increased TRAIL-induced apoptosis, in particular when combined with MG132.^27^ Moreover, the sensitising effect of proteasome inhibition to TRAIL in lymphoma cells with acquired TRAIL resistance could be partially reproduced by knockdown of XIAP.^46^ These findings suggest that XIAP partially accounts for the resistance of HPV^+^ cells to TRAIL as a single treatment. In addition to cytochrome *c*, several other factors are released into the cytosol upon activation of the mitochondrial pathway of apoptosis, including Smac/DIABLO that neutralises the activity of XIAP.^47^ Bortezomib-induced permeabilisation of the outer mitochondrial membrane might therefore result in reduced activity of XIAP to facilitate the full processing of caspase-3 in response to TRAIL stimulation and induction of apoptosis in HPV^+^ cells.

In contrast to many other HNSCCs, HPV-associated tumours retain wild-type p53 that is inactivated by proteasomal degradation through E6 and AP.^35^ Restoration of p53 expression following proteasome inhibition or E6 downregulation was shown to trigger cell cycle arrest and apoptosis in HPV^+^ HNSCC cells.^4,36^ In addition, sensitisation of HPV^+^ cervical cancer cells to TRAIL by proteasome inhibition involved upregulation of p53.^27,48^ Induction of p53 after treatment with chemotherapeutic agents can increase TRAIL sensitivity because of the upregulation of death receptors DR4 and DR5.^49^ Although we observed bortezomib-induced stabilisation of p53 in all HPV^+^ cell lines tested, increased levels of p53 following depletion of E6 were insufficient to sensitise cells to TRAIL. Similar results were obtained previously in HPV-associated cervical cancer cells where transient downregulation of E6 did not confer sensitivity to death receptor-mediated apoptosis.^48^ Our data therefore do not support a role for the E6 oncoprotein in causing TRAIL resistance. Additionally, stabilisation of p53 by bortezomib is unlikely to be responsible for sensitisation to TRAIL.

A recent study suggests that TRAIL can exert enhanced cytotoxic activity in cells arrested in G1 or G2 phase.^50^ Here, bortezomib alone induced clear G2/M arrest in all HPV^+^ and HPV^−^ HNSCC cell lines, which might contribute to their increased sensitivity to TRAIL. While these data point to a potential role for bortezomib-mediated cell cycle arrest, the precise mechanism of sensitisation to TRAIL remains to be determined in future studies.

In conclusion, this study shows that the combination of TRAIL and bortezomib efficiently induces apoptosis through both the death receptor and mitochondrial pathway in TRAIL-resistant HPV^+^ HNSCC cells and enhances TRAIL-mediated cell death in HPV^−^ cells. This combination treatment might therefore represent a novel therapeutic option for drug-resistant HPV^+^ head and neck cancers. However, further investigations, including a larger panel of cell lines and *in vivo* studies, are required to warrant future clinical application of these compounds for the treatment of head and neck cancer patients.

## Materials and Methods

### Cell lines and reagents

Five HPV^+^ and two HPV^−^ cell lines were obtained from various sources and tested for their respective HPV status (Supplementary Table 1). The HPV^−^ cell lines UPCI:SCC089 (089) and UPCI:SCC072 (072), as well as the HPV^+^ cell lines UPCI:SCC090 (090), UPCI:SCC152 (152) and UPCI:SCC154 (154), were a gift from Professor Susanne Gollin, University of Pittsburgh (Pittsburgh, PA, USA). The HPV^+^ cell lines UD-SCC2 (UD2) and VU147-T (147 T) were provided by Professor Henning Bier, University of Munich (München, Germany) and Professor Renske Steenbergen, VU University Amsterdam (Amsterdam, The Netherlands), respectively. All UPCI cell lines and UD-SCC2 cells were cultured in MEM with Earle's salts supplemented with 10% FBS, 2 mM L-glutamine, 100 *μ*g/ml gentamicin and 1 × MEM non-essential amino acids. VU147-T cells were cultured in DMEM high glucose supplemented with 10% FBS, 50 *μ*g/ml streptomycin, 100 *μ*g/ml penicillin and 1 mM sodium pyruvate (all cell culture reagents were obtained from PAA Laboratories, Pasching, Austria).

Recombinant human isoleucine zipper trimerised TRAIL was provided by Professor Henning Walczak, University College London (London, UK). The pancaspase inhibitor zVad was purchased from Promega (Southampton, UK), the proteasome inhibitor MG132 from Sigma-Aldrich (Poole, UK) and nec-1 from StressMarq (Victoria, BC, Canada). Bortezomib was obtained through the Guy's Hospital Pharmacy (London, UK).

### MTT cell viability assay

Cells were seeded in 96-well plates at a density of 10^4^ cells per well and treated with TRAIL and/or bortezomib after 24 h. For inhibitor studies, cells were pre-treated for 1 h with 20 *μ*M zVad and 50 *μ*M nec-1 was included in the treatment. At the indicated times, 20 *μ*l MTT reagent (5 mg/ml; Calbiochem, Watford, UK) was added, followed by incubation for 2–3 h and addition of 150 *μ*l MTT solubilisation solution (50% dimethylformamide, 0.2% glacial acetic acid, 20 mM HCl, 10% SDS). After incubation overnight, the OD_595_ was measured on a LT-4000 microplate reader (Labtech, Uckfield, UK).

Statistical analysis of the results from at least three independent experiments as indicated was performed using GraphPad Prism 6 software (Graphpad Software Inc., La Jolla, CA, USA) and one- or two-way ANOVA as appropriate and specified. Drug synergy was determined using CompuSyn software (ComboSyn Inc., Paramus, NJ, USA) according to the Chou-Talalay method.^51^

### Western blot analysis

Cells were trypsinised, washed in PBS and resuspended in lysis buffer (2 mM MgCl_2_, 25 mM HEPES, 2 mM EGTA, 0.1% Triton X-100) including protease inhibitors. After 30 min incubation on ice, protein lysates were obtained by centrifugation for 15 min at 16 000 × *g*. Forty to sixty micrograms of protein was used for further immunoblot analysis as described previously.^52^

Cytosolic fractions were isolated as described previously.^53^ Briefly, cells of four confluent 15 cm plates were harvested for each condition, cells were washed two times with PBS and resuspended 1 : 1 in ice-cold IBc buffer (10 mM Tris, pH 7.5, 1 mM EGTA, 200 mM sucrose, plus protease inhibitor cocktail). After 30 min incubation, cells were lysed using a 26 G syringe until ~60% of cells were Trypan blue positive. Several centrifugation steps were performed at 700 to 1200 × *g* for 5 min at 4 °C to remove intact cells. The supernatant was centrifuged at 16 000 × *g* for 30 min at 4 °C to obtain the cytosolic/ER fraction of which 100 *μ*g was used for immunoblot analysis.

Antibodies used for immunoblotting were: *β*-actin, tubulin (Sigma-Aldrich), caspase-3, caspase-9, PARP, Bid, p21 (Cell Signalling, Danvers, MA, USA), caspase-8 (Enzo Life Sciences, Exeter, UK), XIAP (BD Transduction Laboratories, Oxford, UK), cytochrome *c* (Abcam, Cambridge, UK) and p53 (Novocastra, Leica Biosystems, Milton Keynes, UK). Secondary HRP-coupled anti-rabbit and anti-mouse antibodies were obtained from GE Healthcare (Chalfont St. Giles, UK) and Sigma-Aldrich, respectively. The relative expression levels of proteins were normalised to *β*-actin using ImageJ software (NIH, Bethesda, MD, USA).

### Flow cytometry

Cells were cultured on 24-well plates at a density of 5 × 10^4^ cells per well and treated with the indicated drugs the next day. Cells were collected at the specific time points by trypsinisation. Apoptosis was quantified by staining with Annexin V-FITC (BD Pharmingen, Oxford, UK) and propidium iodide (PI; Sigma-Aldrich). For cell cycle analysis, cells were fixed in 70% ethanol at −20 °C and stained with DNA staining solution containing PI and RNase A (Sigma-Aldrich) for 30 min. All data were acquired on a BD FACSCanto II cytometer (Beckton-Dickinson, Oxford, UK) and analysed using FlowJo software (Tree Star Inc., Ashland, OR, USA).

For measurement of death receptor expression cells were cultured on 6-well plates at a density of 3 × 10^5^ cells per well and treated with bortezomib the next day. Cells were collected by scraping in PBS and centrifuged for 5 min at 1000 × *g* and 4 °C. Cell pellets were incubated with primary antibodies HS101 (TRAIL-R1, provided by Professor Henning Walczak, University College London, London, UK) and HS201 (TRAIL-R2) or an isotype control (mouse IgG; Sigma-Aldrich) at a concentration of 10 *μ*g/ml for 30 min on ice. Following washing with ice-cold FACS buffer (4% FBS in PBS), a secondary anti-mouse FITC-coupled antibody (Sigma-Aldrich) was added and cells were incubated for 20 min on ice. Samples were centrifuged for 2 min at 800 × *g* and the pellet was resuspended in FACS buffer before acquisition of data on a BD FACSCanto II flow cytometer.

### Lentiviral shRNA knockdown

Lentiviral vectors were produced in HEK293T cells transfected with the second-generation packaging plasmid pCMVΔ8.91 and plasmid pMDG encoding VSV-G-pseudotyped envelope, as well as the construct of interest by calcium phosphate precipitation. At 24, 36 and 48 h after transfection, viral supernatants were harvested, filtered through a 0.45 *μ*m filter and stored at −70 °C supplemented with 5 *μ*g/ml polybrene. After transduction of target cells with the lentivirus, infected cells were selected in the presence of appropriate antibiotics.

A mixture of five Bid shRNA constructs including a non-silencing control shRNA were kindly provided by Professor Simone Fulda, University Hospital Frankfurt (Frankfurt am Main, Germany) (Supplementary Table 2). The XIAP shRNA as well as LacZ shRNA constructs were obtained from Professor Hamid Kashkar, University of Cologne (Köln, Germany).^54^ Inducible caspase-8 and respective scrambled shRNA vectors were provided by Professor Pascal Meier, Institute of Cancer Research (London, UK) (Supplementary Table 2). The constructs for E6 and corresponding scrambled control shRNA were purchased from Santa Cruz (Heidelberg, Germany; sc-156008).

## Abbreviations

TRAIL: tumour necrosis factor-related apoptosis-inducing ligand
HNSCC: head and neck squamous cell carcinoma
HPV^+/−^: human papillomavirus positive/negative
RT/CT: radio-chemotherapy
TRAIL-R: TRAIL receptor
DISC: death-inducing signalling complex
nec-1: necrostatin-1
