# Supplementary material for: Bortezomib sensitises TRAIL-resistant HPV-positive head and neck cancer cells to TRAIL through a caspase-dependent, E6-independent mechanism
Source: Cell Death Dis. 2014 Oct 23;5(10):e1489–. doi: 10.1038/cddis.2014.455 (PMC4649534; doi:10.1038/cddis.2014.455)
Supplement: Supplementary Table 1 [file cddis2014455x1.doc]

**Supplementary Table 1 – Characteristics of HPV Positive and Negative HNSCC Cell Lines.**

| Cell line | HPV | Age | Sex | Site | TNM | Stage | Differentiation | Source |
| --- | --- | --- | --- | --- | --- | --- | --- | --- |
| SCC072 | neg | 61 | F | Tonsil | T3 N2b | IV | M | Prof. S. Gollin  University of Pittsburgh |
| SCC089 | neg | 58 | M | Tonsil | T4 N2b | IV | M | Prof. S. Gollin  University of Pittsburgh |
| SCC090 | pos | 46 | M | BOT | T2 N0 | II | P | Prof. S. Gollin  University of Pittsburgh |
| 147T | pos | 58 | M | FOM | T4 N2 | IV | M | Prof. R. Steenbergen  VU University Amsterdam |
| SCC152* | pos | 47 | M | HP | Unknown | NA | M | Prof. S. Gollin  University of Pittsburgh |
| SCC154 | pos | 54 | M | T | T4 N2 | IV | P | Prof. S. Gollin  University of Pittsburgh |
| UD-SCC2 | pos |  |  | Hypopharynx | T3 N3 | IV |  | Prof. H Bier  University of Munich |

^a^T = tongue, BM = buccal mucosa, BOT = base of tongue, FOM = floor of mouth, AP = alveolar process, HP=hypopharynx.

^b^W = well differentiated; M = moderately differentiated; P= poorly differentiated

*Recurrence of SCC090
