# Supplementary material for: Bortezomib sensitises TRAIL-resistant HPV-positive head and neck cancer cells to TRAIL through a caspase-dependent, E6-independent mechanism
Source: Cell Death Dis. 2014 Oct 23;5(10):e1489–. doi: 10.1038/cddis.2014.455 (PMC4649534; doi:10.1038/cddis.2014.455)
Supplement: Supplementary Table 2 [file cddis2014455x2.doc]

**Supplementary Table 2 – Targeting Sequences of shRNA Constructs.**

| shBid 1 (RHS4430-99157271) | TGCTGTTGACAGTGAGCGCG**GCCACTGTTTGGAAATAAA**TTAGTGAAGCCACAGATGTAA**TTTATTTCCAAACAGTGGC**CTTGCCTACTGCCTCGGA |
| --- | --- |
| shBid 2 (RHS4430-101159294) | TGCTGTTGACAGTGAGCGCC**TGGTGTTTGGCTTCCTCCA**ATAGTGAAGCCACAGATGTAT**TGGAGGAAGCCAAACACCA**GTTGCCTACTGCCTCGGA |
| shBid 3 (RHS4430-101159724) | TGCTGTTGACAGTGAGCGAT**CCGTGATGTCTTTCACACA**ATAGTGAAGCCACAGATGTAT**TGTGTGAAAGACATCACGG**AGTGCCTACTGCCTCGGA |
| shBid 4 (RHS4430-101162430) | TGCTGTTGACAGTGAGCGAC**TCGATGTGGTCACAGCTGT**ATAGTGAAGCCACAGATGTAT**ACAGCTGTGACCACATCGA**GCTGCCTACTGCCTCGGA |
| shBid 5 (RHS4430-101169631) | TGCTGTTGACAGTGAGCGCC**AGGGATGAGTGCATCACAA**ATAGTGAAGCCACAGATGTAT**TTGTGATGCACTCATCCCT**GATGCCTACTGCCTCGGA |
|  |  |
| sh Caspase-8 (1213) | TGCTGTTGACAGTGAGCGCC**TGCACAGTAGAGCAAATCT**ATAGTGAAGCCACAGATGTA**TAGATTTGCTCTACTGTGCA**GTTGCCTACTGCCTCGGA |
