# Supplementary material for: Bortezomib sensitises TRAIL-resistant HPV-positive head and neck cancer cells to TRAIL through a caspase-dependent, E6-independent mechanism
Source: Cell Death Dis. 2014 Oct 23;5(10):e1489–. doi: 10.1038/cddis.2014.455 (PMC4649534; doi:10.1038/cddis.2014.455)
Supplement: Supplementary Figure 1 [file cddis2014455x3.ppt]

## Slide 1
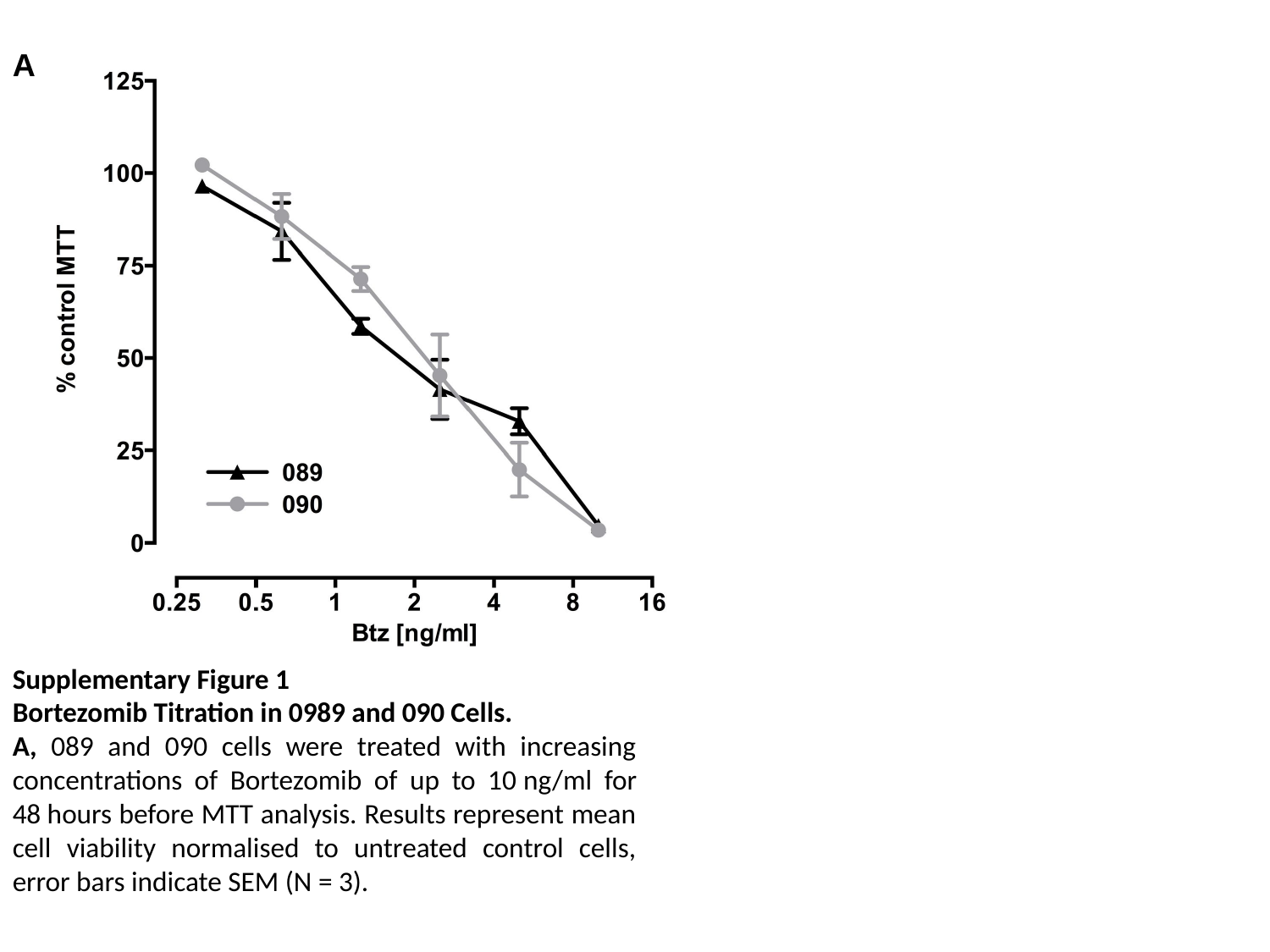

A
Supplementary Figure 1
Bortezomib Titration in 0989 and 090 Cells.
A, 089 and 090 cells were treated with increasing concentrations of Bortezomib of up to 10 ng/ml for 48 hours before MTT analysis. Results represent mean cell viability normalised to untreated control cells, error bars indicate SEM (N = 3).
