# Supplementary material for: Bortezomib sensitises TRAIL-resistant HPV-positive head and neck cancer cells to TRAIL through a caspase-dependent, E6-independent mechanism
Source: Cell Death Dis. 2014 Oct 23;5(10):e1489–. doi: 10.1038/cddis.2014.455 (PMC4649534; doi:10.1038/cddis.2014.455)
Supplement: Supplementary Figure 2 [file cddis2014455x4.ppt]

## Slide 1
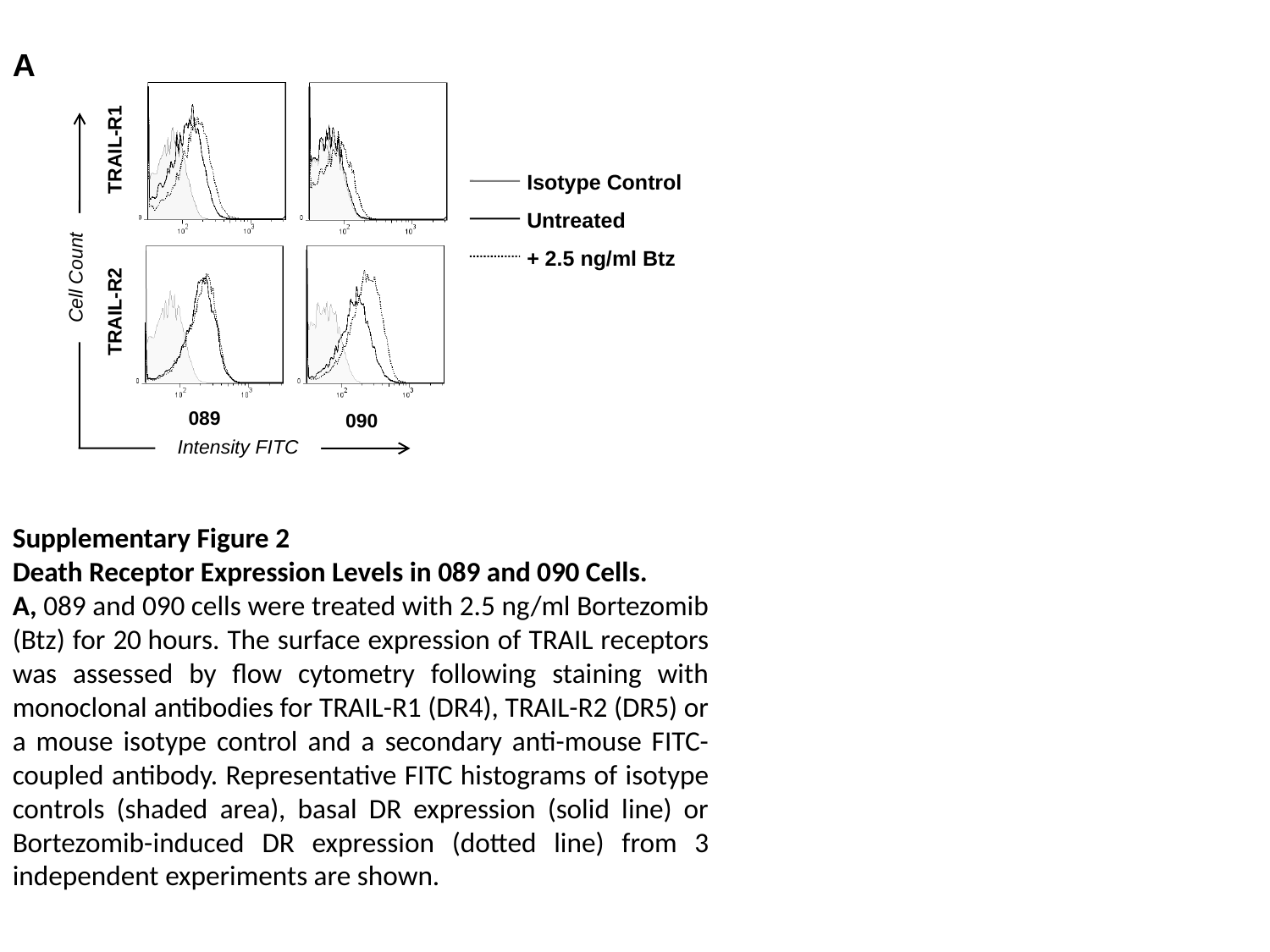

A
TRAIL-R1
Cell Count
TRAIL-R2
089
090
Intensity FITC
Isotype Control
Untreated
+ 2.5 ng/ml Btz
Supplementary Figure 2
Death Receptor Expression Levels in 089 and 090 Cells.
A, 089 and 090 cells were treated with 2.5 ng/ml Bortezomib (Btz) for 20 hours. The surface expression of TRAIL receptors was assessed by flow cytometry following staining with monoclonal antibodies for TRAIL-R1 (DR4), TRAIL-R2 (DR5) or a mouse isotype control and a secondary anti-mouse FITC-coupled antibody. Representative FITC histograms of isotype controls (shaded area), basal DR expression (solid line) or Bortezomib-induced DR expression (dotted line) from 3 independent experiments are shown.
