# Supplementary material for: Bortezomib sensitises TRAIL-resistant HPV-positive head and neck cancer cells to TRAIL through a caspase-dependent, E6-independent mechanism
Source: Cell Death Dis. 2014 Oct 23;5(10):e1489–. doi: 10.1038/cddis.2014.455 (PMC4649534; doi:10.1038/cddis.2014.455)
Supplement: Supplementary Figure 3 [file cddis2014455x5.ppt]

## Slide 1
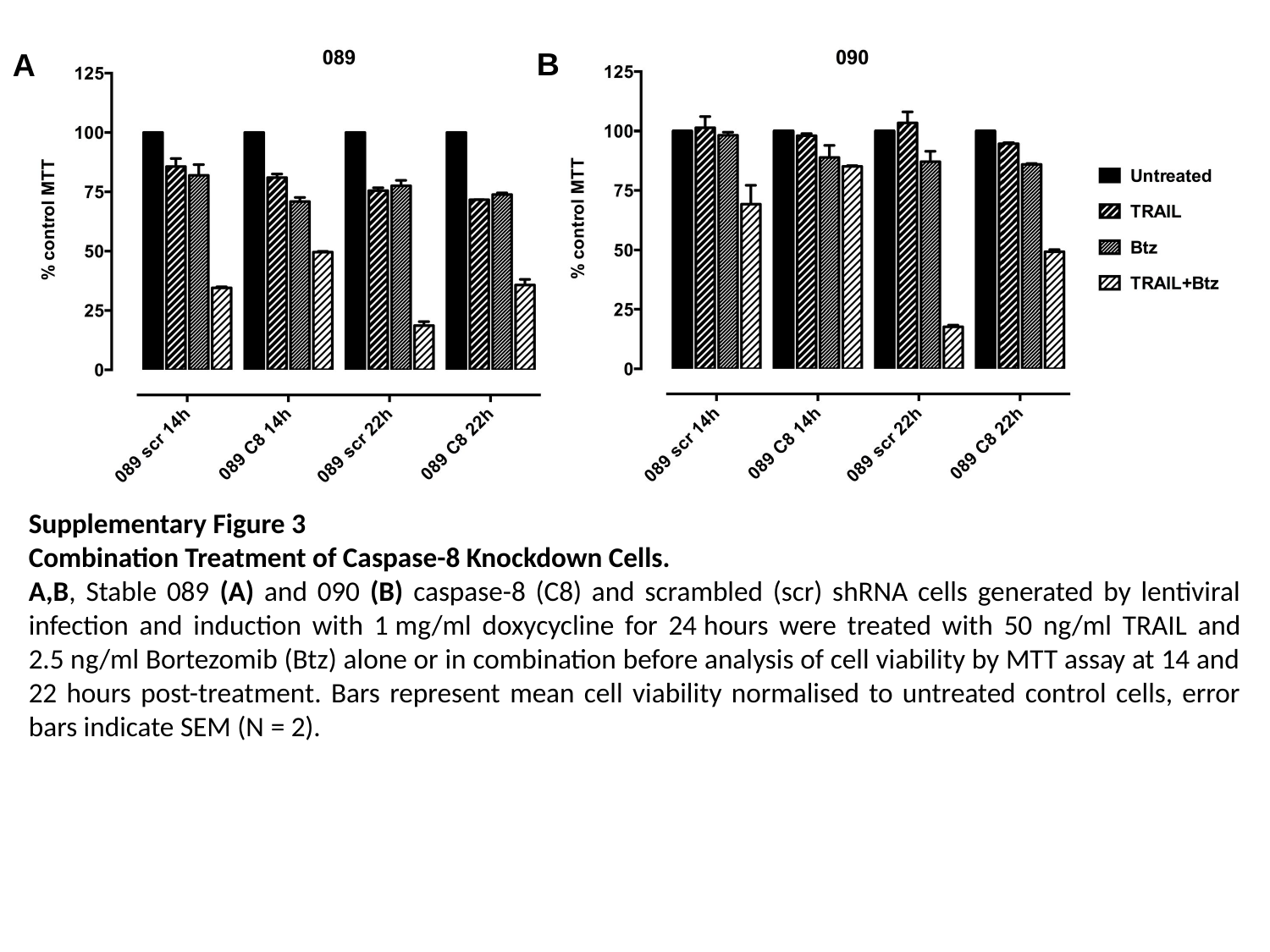

B
A
Supplementary Figure 3
Combination Treatment of Caspase-8 Knockdown Cells.
A,B, Stable 089 (A) and 090 (B) caspase-8 (C8) and scrambled (scr) shRNA cells generated by lentiviral infection and induction with 1 mg/ml doxycycline for 24 hours were treated with 50 ng/ml TRAIL and 2.5 ng/ml Bortezomib (Btz) alone or in combination before analysis of cell viability by MTT assay at 14 and 22 hours post-treatment. Bars represent mean cell viability normalised to untreated control cells, error bars indicate SEM (N = 2).
